# Accrual of Alzheimer's disease pathology as a function of proximity to parental dementia onset

**DOI:** 10.1002/dad2.70092

**Published:** 2025-02-27

**Authors:** Elina T. Ziukelis, Elijah Mak, Craig Ritchie, John T. O'Brien, Dag Aarsland, for the European Prevention of Alzheimer's Disease (EPAD) Consortium

**Affiliations:** ^1^ Institute of Psychiatry, Psychology & Neuroscience at King's College London London UK; ^2^ South London & Maudsley NHS Foundation Trust London UK; ^3^ Department of Psychiatry, School of Clinical Medicine University of Cambridge, Level E4 Cambridge Biomedical Campus Cambridge UK; ^4^ Scottish Brain Sciences, Gyleview House Edinburgh UK; ^5^ Edinburgh Dementia Prevention, Centre for Clinical Brain Sciences University of Edinburgh Edinburgh UK; ^6^ School of Medicine University of St Andrews St Andrews UK

**Keywords:** APOE‐𝜀 genotype, estimated years to onset, late‐onset Alzheimer's disease, parental age at onset, preclinical Alzheimer's disease, proximity to parental onset, sporadic Alzheimer's disease

## Abstract

**INTRODUCTION:**

Whether temporal proximity to parental onset of dementia (PPO) can be used to estimate timing of the preclinical stage of sporadic Alzheimer's disease (AD) remains uncertain.

**METHODS:**

We investigated cross‐sectionally adults aged > 50 without dementia included in the European Prevention of Alzheimer's Dementia (EPAD) study. PPO was tested as a predictor of quantitative levels of cerebrospinal fluid (CSF) β‐amyloid (1‐42) (Aβ1‐42) in those with a parental history of dementia (*n* = 688) and of phosphorylated tau (p‐tau) and EPAD neuropsychological examination (ENE) subscores in an amyloid positive subgroup (*n* = 226). Possible interactions were explored.

**RESULTS:**

Shorter PPO predicted lower CSF Aβ1‐42 level (β = 9.357; T = 4.161; *p* < 0.001), interacting with apolipoprotein E (APOE) ‐𝜀4 carriage in a dose‐dependent manner. Concomitant APOE‐𝜀2 carriage appeared to provide protection. PPO did not predict p‐tau levels or cognitive performance.

**DISCUSSION:**

PPO may provide a valid method of stratifying risk of early AD pathologic change in APOE‐𝜀4 carriers, with empirical and clinical applications.

**Highlights:**

- Proximity to age of parental dementia onset can predict amyloid accrual
- The effect is APOE‐𝜀4 dose‐dependent and APOE‐𝜀2 appears to provide protection
- PPO does not appear to predict further advancement along the AD continuum
- In the era of anti‐amyloid treatments, this may inform timing of amyloid screening
- Used as an empirical metric, PPO could help elucidate the natural history of LOAD

## 1 BACKGROUND

Alzheimer's dementia comprises the terminal phase of a protracted and nonlinear sequence of neuropathological changes associated with Alzheimer's disease (AD).^1^, ^2^ Improved understanding of the preclinical disease course is imperative to further development and trial of disease‐modifying treatments. AD risk‐enriched study cohorts yet to develop dementia provide a valuable resource.

The concept of “estimated years to symptom onset” (EYO) has been helpfully applied to the study of presymptomatic familial AD, taking advantage of the high penetrance of autosomal dominant mutations and consistency in age of symptom onset within affected families. Age of symptom onset in an individual mutation carrier can be predicted by both mean age of onset among carriers of the specific mutation and parental age of symptom onset.^3^ Though the former is a stronger correlate, EYO can be meaningfully derived from either. Early studies of presymptomatic mutation carriers utilizing EYO as an experimental metric^4^, ^5^ helped elucidate the timing and sequence of pathological changes during the preclinical phase,^1^ justifying subsequent use as a proxy stage marker.^6^, ^7^, ^8^

Validity of EYO as a proxy stage marker in studies of late‐onset AD (LOAD) is less certain, as genetic contributions are more complex. However, there is evidence that timing as well as overall risk of LOAD is highly heritable. One multicenter study of 4302 probands suggested heritability of age of onset to be 66.9%–86.8%.^9^ Genomic studies have indicated that this is largely driven by apolipoprotein E (APOE) genotype.^10^, ^11^

Consistent with high heritability of age of onset, nearness to parental age of LOAD has been associated with structural and functional neuroimaging changes^12^, ^13^ and cognitive decline^14^ independently of age. In the case of white matter microstructural differences in a cohort aged 40–59 years, comparison of groups “near” and “far” to parental dementia onset produced larger effect sizes than comparisons based on age or APOE‐𝜀4 carriage.^13^ This suggests it could be a more powerful risk stratification tool in studies of LOAD, capable of highlighting subtle incipient pathology.

Few studies have sought to confirm a relationship between years to parental onset of LOAD and established AD‐specific biomarkers. It has been shown that proximity to parental onset predicts accrual of amyloid pathology cross‐sectionally^15^, ^16^ and longitudinally.^16^ However, interactions with gender^15^, ^16^, age,^15^ and APOE‐𝜀4 carriage^16^ have been inconsistent across relatively small (*n* < 300) datasets and amyloid measures. Based on a single study of asymptomatic individuals aged 49–73 years,^15^ years to parental dementia onset does not predict levels of CSF p‐tau, CSF t‐tau, or hippocampal volume.

**RESEARCH IN CONTEXT**

1. **Systematic review**: The authors searched database PubMed for articles describing experimental use of a measure of proximity to parental onset of dementia (PPO). We cite studies of preclinical Alzheimer's Disease (AD) which have used the metric as a risk stratification tool and highlight preliminary evidence substantiating its use by association with established biomarkers.
2. **Interpretation**: Results reveal a specific population for whom PPO could be used to estimate the timing of early Alzheimer's pathologic change. As well as substantiating application of the metric to APOE‐𝜀4+ individuals in studies of late onset AD, this has direct clinical applications.
3. **Future directions**: Selective examination of APOE‐𝜀4+ individuals as they approach age of parental dementia onset could elucidate: a) novel biomarkers paralleling or preceding amyloid accrual b) factors predicting evasion of amyloid pathology despite escalating risk conferred by PPO and c) factors moderating the timing of subsequent progression to tauopathy and cognitive impairment.

It is possible that heritability of the timing of accrual of AD pathology is higher than that of symptom onset. A myriad of well‐established genetic, epigenetic, and environmental risk and protective factors^17^ conceivably moderate an individual's progression along the AD continuum. Therefore, we adopt the term *proximity to parental onset* (PPO). PPO describes the time remaining until an individual reaches their parent's age of dementia onset without suggesting its implications.

In the current study, we aim to better inform use of PPO as a risk stratification tool in studies of LOAD by clarifying its relationship to measures utilized in A/T/N staging.^18^ We utilize power conferred by a considerably larger cohort than those analyzed within previous studies to: (1) replicate an association between PPO and CSF Aβ1‐42^16^; (2) Determine whether PPO predicts CSF p‐tau or cognitive performance in those evidenced to be in the preclinical phase of AD by low CSF Aβ1‐42; (3) explore in unprecedented detail potential interactions with PPO in prediction of CSF Aβ1‐42, CSF p‐tau, and neuropsychological evaluation scores; and (4) where there is an association between PPO and a CSF marker for AD pathology, quantify progression of the risk of testing positive as age of parental dementia onset approaches.

## 2 METHODS

### 2.1 Participants and study design

This study used baseline data from the prospective, multicenter European Prevention of Alzheimer's Dementia (EPAD) Longitudinal Cohort Study (LCS). EPAD LCS is registered at www.clinicaltrials.gov Identifier: NCT02804789. Data used in preparation of this article were obtained from the EPAD LCS data set V1500.0, doi:10.34688/epadlcs_v1500.0_19.11.29. The EPAD LCS was launched in 2015 as a public‐private partnership. The primary research goal of the EPAD LCS is to provide a well‐phenotyped probability‐spectrum population for developing and continuously improving disease models for AD in individuals without dementia. The protocol has been previously published.^19^

Participants were recruited from parent cohorts throughout Europe as well as clinical settings. Exclusion criteria included CDR score > 0.5, known carriage of a PSEN1, PSEN2, or APP mutation associated with autosomal dominant AD and other neurodegenerative conditions. Only those with a reported history of dementia in one or more biological parents and who underwent lumbar puncture were included in the current study (*n* = 688).

### 2.2 Ethics

All procedures complied with the World Medical Association Declaration of Helsinki. Protocol and materials were approved by the Independent Ethics Committees local to each study site. The study received ethical approval from numerous institutional review boards across Europe.

2.3

Participants self‐reported parental history of AD including age at diagnosis. Biological relatedness was clarified. Acknowledging public misperception about the difference between AD and other dementias, limitations of current clinical diagnostic tools and likelihood that data included other dementia subtypes, we refer to onset of parental dementia rather than AD. PPO was calculated by subtracting a subject's age from the age at which a biological parent was diagnosed. A negative value therefore indicates that the subject had surpassed the age of parental dementia diagnosis. For those with two affected parents, the earliest age was selected.

### 2.4 CSF measures

CSF samples were analyzed using the Roche cobas Elecsys System, yielding concentrations of Aβ1‐42, p‐tau181, and t‐tau. The assays had lower detection limits for Aβ1‐42 and p‐tau of < 200 and < 8 pg/mL respectively. Values recorded as < 200 or < 8 pg/mL were assigned values of 200 and 8 pg/mL, respectively. Levels of Aβ1‐42 exceeding 1700 pg/mL were assigned a value of 1700 pg/mL, as performance of the assay beyond this upper limit has not been established. Cutoff values of < 1000 pg/mL for Aβ1‐42 positivity and > 27 pg/mL for p‐tau positivity were validated by Ingala and colleagues using Gaussian mixture models.^20^ t‐Tau levels were not utilized in the current study (see Supplementary Methods).

### 2.5 APOE genotype

Taqman genotyping was performed on DNA extracted from whole blood samples to determine subjects’ specific APOE variants. These data were available for 654 participants. Three variables were created: (1) a three‐factor variable (“APOE‐𝜀4 status”) consisting of APOE‐𝜀4 homozygote, APOE‐𝜀4 heterozygote, and APOE‐𝜀4 negative groups; (2) a two‐factor variable (“APOE‐𝜀2 status”) consisting of APOE‐𝜀2 positive and negative groups; and (3) a six‐factor variable (“Allele Combination”) separating subjects by precise APOE‐𝜀 allele combination as follows: 2/2, 2/3, 2/4, 3/3, 3/4, and 4/4.

### 2.6 Family history load and risk inheritance

A two‐factor variable (family history load) was created based on the number of biological parents who had been diagnosed with dementia for each subject (1 or 2). A three‐factor variable (risk inheritance) was created based on gender of the biological parent who had been diagnosed (maternal inheritance, paternal inheritance, or both).

### 2.7 Cognitive testing

The Clinical Dementia Rating (CDR) Scale was used to define subjects as either cognitively normal (CDR = 0) or mildly cognitively impaired (MCI) (CDR = 0.5). Additional cognitive testing included the EPAD Neuropsychological Examination (ENE).^21^, ^22^ The current study used a single subscore from each of 13 cognitive tests within the ENE. A list of subscores together with the number of subjects for which they were available (range 112–225) is included in supplementary material (see Supplementary Methods).

### 2.8 Statistical analyses

All statistical analyses were conducted in R (version 2023.09.1+494). In three separate sets of analyses, robust linear regression was used to determine whether PPO predicts: (1) CSF Aβ1‐42 in the whole sample; (2) CSF p‐tau in amyloid‐positive subjects; and (3) 13 individual ENE subscores in amyloid‐positive subjects.

Amyloid‐positive subjects were chosen for the second and third sets of analyses in an effort to isolate subjects evidencing “Alzheimer's pathologic change” and therefore demonstrably on the AD spectrum.^23^ There is substantial biomarker modeling evidence to suggest that reduction in CSF Aβ1‐42 precedes both increase in CSF p‐tau and cognitive impairment during the AD course.^1^ In addition, approximately 10% of the EPAD cohort were previously shown to meet thresholds for tau and/or neurodegeneration but not amyloid positivity.^20^ We considered that experimental noise created by inclusion of individuals accruing tau in the absence of amyloid may obscure an association relevant specifically to AD.

Within each set of analyses, three linear regression models were tested. First, to determine if a subject's age alone could predict them, each dependent variable was regressed on age including gender and education level as covariates. Second, to determine if PPO could predict them while accounting for the effect of age, each dependent variable was regressed on PPO including age as well as gender and education level as covariates. Third, potential interactions with PPO in prediction of the dependent variables were tested, including age, gender, and education level as covariates. The following potential interactions were sequentially tested: age; gender; education; APOE‐𝜀4 status; APOE‐𝜀2 status; family history load; risk inheritance; and CDR total score.

Within the third set of analyses, 13 ENE subscores were assessed in turn as dependent variables. Therefore, for each model, in this set correction for multiple comparisons was made using the Benjamin & Hochberg method. For all tests, statistical significance was considered when *p* < 0.05 (post correction where applicable).

Use of linear regression was justified based on overall results of analysis of variance (ANOVA) comparisons of linear and non‐linear regression models for all three sets of analyses. Variance inflation factor analyses were also performed to test collinearity between age and PPO. These suggested that PPO and age were not strongly correlated.

Finally, progression of the risk of amyloid positivity and of tau positivity as age of parental dementia onset approached in the study sample was quantified. In order to do this, the sample was divided into six groups based on PPO (> 20 years [*n* = 38], 16–20 years [*n* = 53], 11–15 years [*n* = 67], 6–10 years [*n* = 38], 0–5 years [*n* = 36], and < 0 years [*n* = 23]). The proportion of each group testing positive was calculated and plotted to show how PPO might be used to predict likelihood of pathology in practice.

## 3 RESULTS

### 3.1 Demographics

Demographic details for the whole baseline study sample are summarized in Table 1. Group differences between APOE‐*𝜀*4 negative subjects, APOE‐*𝜀*4 heterozygotes, and APOE‐*𝜀*4 homozygotes are shown in Table 2. The higher likelihood of MCI in homozygotes despite age‐matched groups is consistent with a typically younger age of onset.^24^

**TABLE 1:** Demographics (whole sample, *n* = 688).

Characteristic	*n* = 688^a^
**Age (years)**	
*Mean (SD)*	63.74 (6.80)
*Range*	50.00, 87.00
**Gender**	
*Female*	422 (61%)
*Male*	266 (39%)
**Education (years)**	
*Mean (SD)*	14.74 (3.65)
*Range*	7.00, 28.00
**CDR Total**	
*Normal (CDR = 0)*	599 (87%)
*MCI (CDR = 0.5)*	88 (13%)
**PPO diagnosis**	
*Mean (SD)*	12.65 (9.12)
*Range*	−29.00, 35.00
**FH of dementia**	
*FH+ (1 parent)*	612 (89%)
*FH++ (2 parents)*	76 (11%)
**Risk inheritance**	
*Maternal*	445 (65%)
*Paternal*	167 (24%)
*Both parents*	76 (11%)
**APOE‐*𝜀* Allele combination**	
*2/2*	1 (0.2%)
*3/3*	324 (50%)
*4/4*	30 (4.6%)
*3/4*	225 (34%)
*2/3*	49 (7.5%)
*2/4*	25 (3.8%)

*Note*: Participant characteristics of subjects for whom age of parental dementia onset and CSF Aβ1‐42 and p‐tau were available. PPO was defined as the difference between a subject's age and their parent's age at dementia diagnosis, selecting the earliest age for those with two affected parents. A negative value indicates that the subject had surpassed the age of parental dementia diagnosis.

Abbreviations: APOE, apolipoprotein E; CDR, Clinical Dementia Rating Scale; FH, family history; PPO, proximity of parental onset of dementia; SD, standard deviation.

^a^ *n*(%).

**TABLE 2:** Demographics by APOE‐𝜀4 status (*n* = 654).

	APOE‐*𝜀*4 status				
Characteristic	Overall, *n* = 654^a^	APOE‐*𝜀*4‐, *n* = 374^a^	APOE‐*𝜀*4+, *n* = 250^a^	APOE‐*𝜀*4++, *n* = 30^a^	*p* ^b^
**Age (years)**					0.65
*Mean (SD)*	63.72 (6.81)	63.88 (7.07)	63.42 (6.39)	64.27 (6.94)	
*Range*	50.00, 87.00	50.00, 87.00	50.00, 83.00	51.00, 76.00	
**Gender**					0.78
*Female*	406 (62%)	235 (63%)	154 (62%)	17 (57%)	
*Male*	248 (38%)	139 (37%)	96 (38%)	13 (43%)	
**Education (years)**					**0.017**
*Mean (SD)*	14.72 (3.62)	14.96 (3.75)	14.52 (3.47)	13.33 (2.87)	
*Range*	7.00, 28.00	7.00, 26.00	7.00, 28.00	9.00, 19.00	
**CDR Total**					**0.042**
*Normal (CDR = 0)*	569 (87%)	321 (86%)	226 (90%)	22 (76%)	
*MCI (CDR = 0.5)*	84 (13%)	53 (14%)	24 (9.6%)	7 (24%)	
**PPO diagnosis**					**0.016**
*Mean (SD)*	12.70 (9.10)	13.40 (8.99)	12.06 (9.06)	9.27 (9.98)	
*Range*	−29.00, 35.00	−18.00, 35.00	−29.00, 35.00	−8.00, 26.00	
**FH of dementia**					0.10
*FH+ (1 parent)*	584 (89%)	342 (91%)	215 (86%)	27 (90%)	
*FH++ (2 parents)*	70 (11%)	32 (8.6%)	35 (14%)	3 (10%)	
**Risk inheritance**					0.29
*Maternal*	424 (65%)	251 (67%)	154 (62%)	19 (63%)	
*Paternal*	160 (24%)	91 (24%)	61 (24%)	8 (27%)	
*Both parents*	70 (11%)	32 (8.6%)	35 (14%)	3 (10%)	

Participant characteristics of subjects grouped by APOE‐𝜀4 Status (APOE‐𝜀4 negative [APOE‐*𝜀*4‐], APOE‐*𝜀*4 heterozygote [APOE‐*𝜀*4+], APOE‐*𝜀*4 homozygote [APOE‐*𝜀*4++]).

Abbreviations: APOE, apolipoprotein E; CDR, Clinical Dementia Rating Scale; FH, family history; PPO, proximity of parental onset of dementia; SD, standard deviation.

*p* < 0.05.

^a^ *n*(%).

^b^ Kruskall‐Wallis rank sum test; Pearson's chi‐squared test; Fisher's exact test.

### 3.2 CSF Aβ1‐42 as a function of PPO

Subject age was a significant predictor of CSF Aβ1‐42 level (β = ‐9.431, T = ‐3.613, *p* < 0.001). However, shorter PPO predicted lower CSF Aβ1‐42 level even when accounting for the effect of age, gender, and education level (β = 9.357; T = 4.161; *p* < 0.001) (see Supplementary Results, Table S1, and Figure S1 for further data facilitating comparison of age and PPO).

**FIGURE 1:**
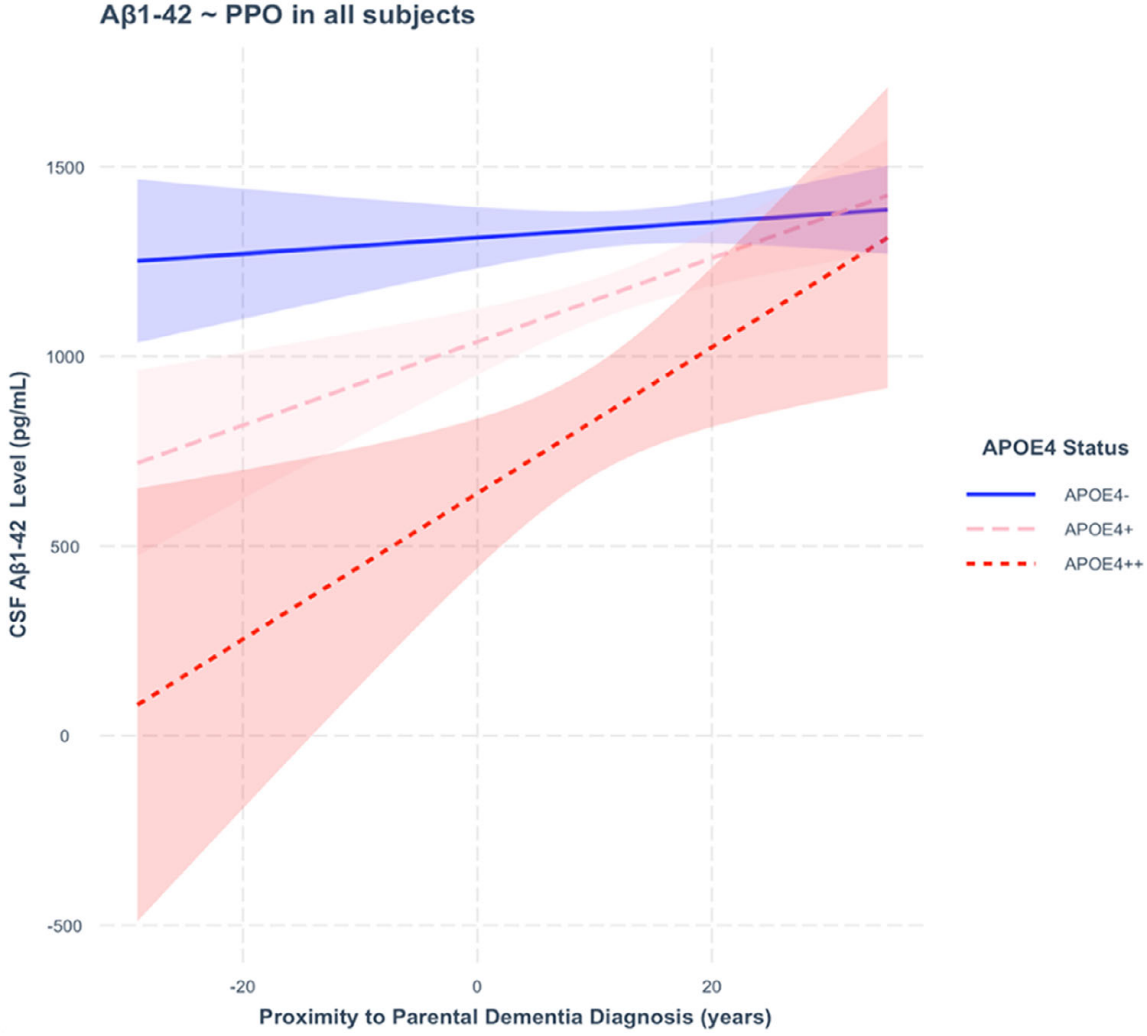
Cerebrospinal fluid (CSF) Aβ1‐42 as a function of proximity to parental onset of dementia (PPO). CSF Aβ1‐42 level in picograms per milliliter plotted against PPO in years, including only subjects for which apolipoprotein E (APOE) ‐*𝜀*4 data were available (*n* = 654). APOE‐*𝜀*4 negativity is denoted by APOE4‐ (in blue), APOE‐*𝜀*4 heterozygosity by APOE4+ (in pink), and APOE‐*𝜀*4 homozygosity by APOE4++ (in red). As subjects approach age of parental dementia onset (x > 0) and then surpass it (x < 0), CSF Aβ1‐42 level decreases. At any given value for PPO, APOE‐*𝜀*4 carriage is associated with lower CSF Aβ1‐42 in a dose‐dependent manner. Shaded areas indicate 95% confidence intervals for CSF marker levels.

PPO interacted significantly with APOE‐𝜀4 carriage to predict CSF Aβ1‐42 level (Figure 1). Compared with APOE‐𝜀4 negativity, both APOE‐𝜀4 heterozygosity (β = 10.498, T = 2.608, *p* = 0.00931) and APOE‐𝜀4 homozygosity (β = 17.283, T = 2.316, *p* = 0.02086) were associated with steeper decline in CSF Aβ1‐42 on approach of age of parental dementia onset. Decline was sharpest for homozygotes (Figure 1). PPO did not significantly interact with age, gender, education, APOE‐𝜀2 status, family history load, risk inheritance, or CDR score to predict CSF Aβ1‐42 level (Table S2).

**FIGURE 2:**
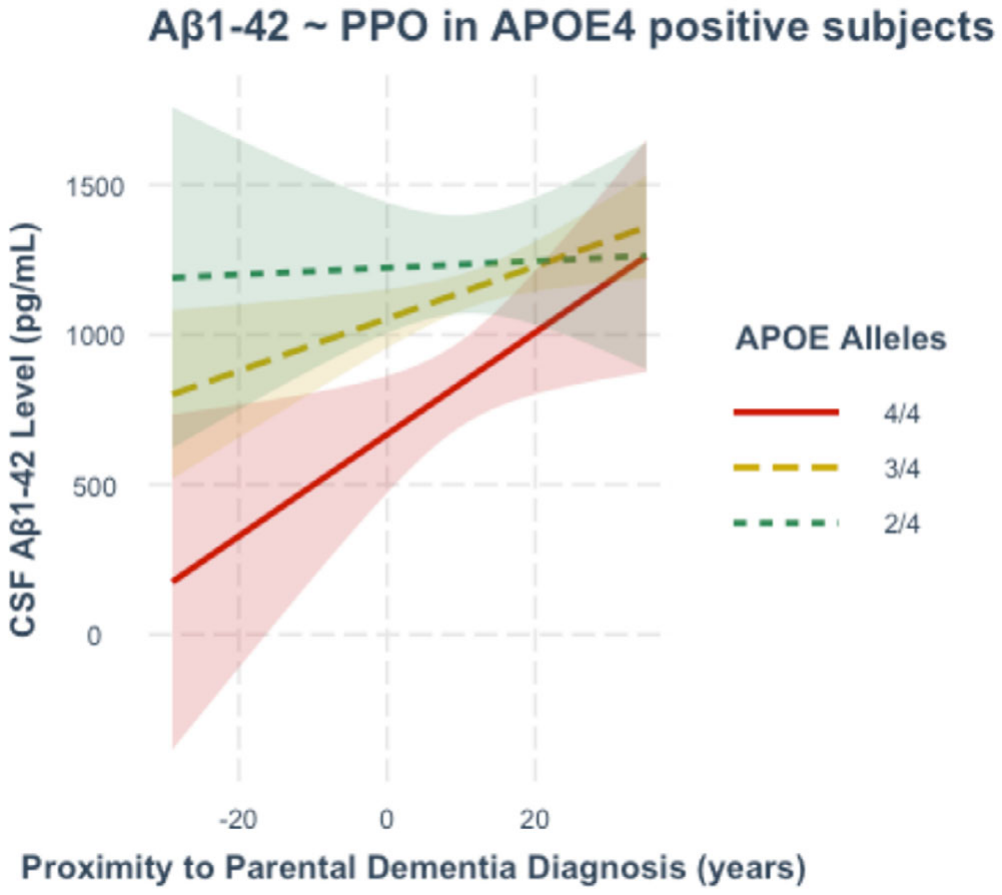
Post‐hoc analyses. Cerebrospinal fluid (CSF) Aβ1‐42 level in picograms per milliliter plotted against proximity to parental onset of dementia (PPO), including only apolipoprotein E (APOE) ‐*𝜀*4 positive subjects (*n* = 280). APOE‐*𝜀* allele combinations are denoted by 4/4 (two APOE‐*𝜀*4 alleles, shown in red), 3/4 (one APOE‐*𝜀*3 allele and one APOE‐*𝜀*4 allele, shown in yellow), and 2/4 (one APOE‐*𝜀*2 allele and one APOE‐*𝜀*4 allele, shown in green). The interaction between PPO and precise APOE‐*𝜀* allele combination did not meet the significance threshold. However, within the APOE‐𝜀4+/ APOE‐𝜀2+ group (*n* = 25), PPO was not a significant predictor of Aβ1‐42 level, suggesting that APOE‐*𝜀*2 provides a protective effect. Shaded areas indicate 95% confidence intervals for CSF marker levels.

Post‐hoc analyses of the APOE‐*𝜀*4 negative group only (*n* = 374) showed that PPO was not a significant predictor of CSF Aβ1‐42 (β = 4.178, T = 2.876, *p* = 0.147), suggesting that the effect in the whole sample is conferred by APOE‐*𝜀*4 carriage. In analyses of the APOE‐*𝜀*4 positive group (*n* = 280), precise APOE‐*𝜀* allele combination appeared graphically to interact with PPO to predict CSF Aβ1‐42 (Figure 2). Compared with the APOE‐*𝜀* allele combination 4/4, the 3/4 (β = ‐7.902, T = ‐1.022, *p* = 0.308) and 4/2 (β = ‐16.872, T = ‐1.911, *p* = 0.057) combinations were not associated with a significantly different trajectory of CSF Aβ1‐42 on approach of parental age of onset of dementia. However, it was noted that the graphically apparent protective effect of APOE‐*𝜀*2 was of borderline significance and the number of subjects carrying 2/4 (*n* = 25) and 4/4 (*n* = 30) combinations was small relative the number carrying 3/4 (*n* = 225). Within the APOE‐𝜀4+/ APOE‐𝜀2+ group (*n* = 25), PPO was not a significant predictor of Aβ1‐42 level (β = ‐4.410, T = ‐0.667, *p* = 0.513).

**FIGURE 3:**
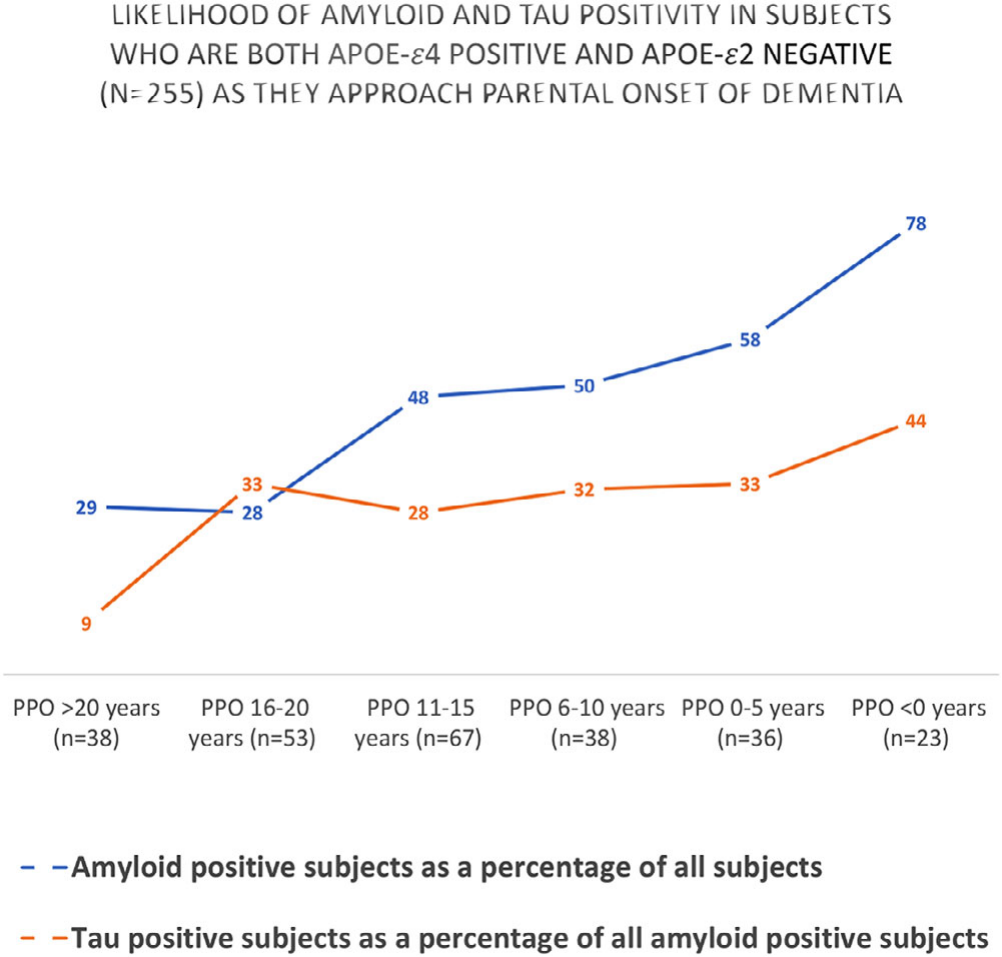
Likelihood of amyloid and tau positivity in apolipoprotein E (APOE) ‐𝜀4 positive, APOE‐𝜀2 negative subjects (*n* = 255) on approach of parental onset of dementia. PPO denotes proximity (in years) to age of parental dementia diagnosis. The percentage of total APOE‐𝜀4+/ APOE‐𝜀2‐ subjects (*n* = 255) meeting criteria for amyloid positivity based on cerebrospinal fluid (CSF) Aβ1‐42 level increased with proximity to age of parental dementia diagnosis. However, the proportion of those who were amyloid positive additionally meeting criteria for p‐tau positivity did not appear to rise concomitantly.

At least half of APOE‐𝜀4+/APOE‐𝜀2‐ subjects (*n* = 255) within 10 years of age of parental onset were amyloid positive, the proportion increasing as age of parental dementia onset is approached and then exceeded (Figure 3). Further post‐hoc analyses were conducted on this group to determine whether lifestyle factors might moderate the relationship between PPO and CSF Aβ1‐42. PPO did not significantly interact with frequency of physical activity, physical fitness level, systolic blood pressure, waist circumference, smoking status, or body mass index to predict CSF Aβ1‐42 level. Age and gender were included as covariates.

Given evidence of lower genetic influence on the development of very late onset AD (VLOAD),^25^ analyses were repeated on a subsample whose parent had been diagnosed with dementia before age 85 (*n* = 579). This did not change results.

### 3.3 CSF p‐tau as a function of PPO

**FIGURE 4:**
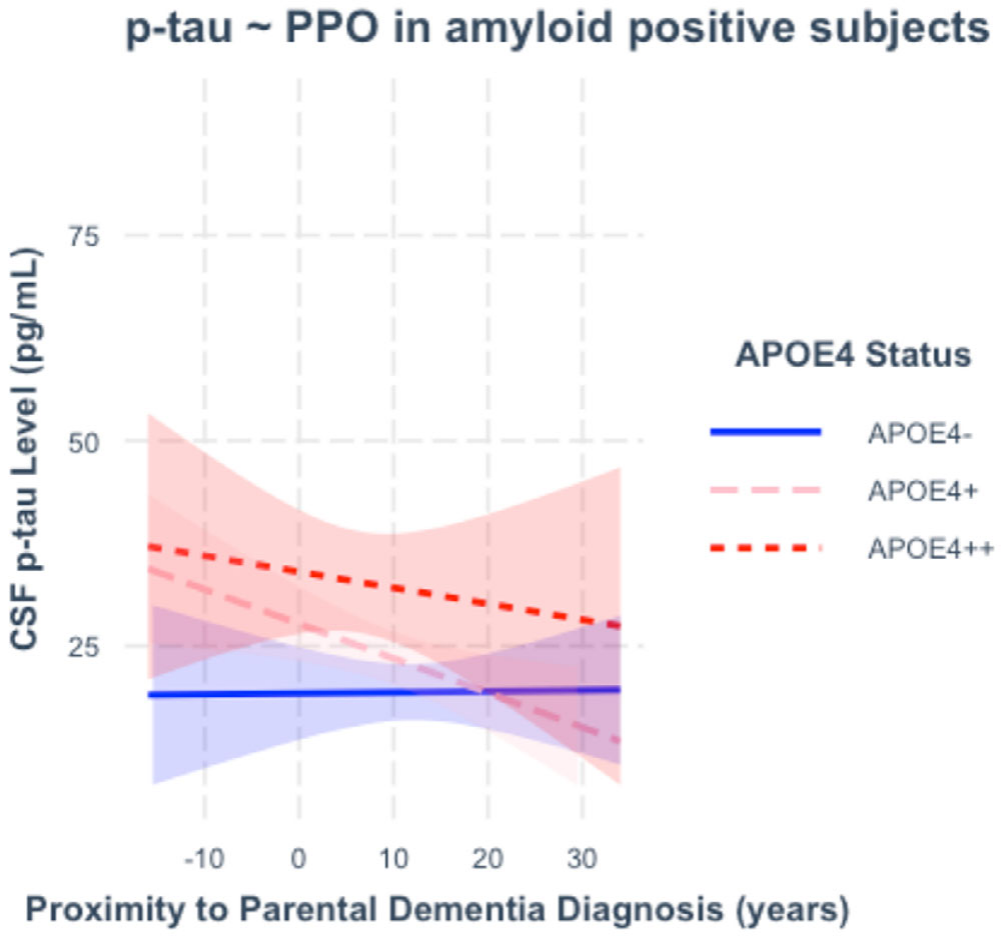
Cerebrospinal fluid (CSF) p‐tau as a function of proximity to parental onset of dementia (PPO) in amyloid positive subjects. Cerebrospinal fluid (CSF) p‐tau level in picograms per milliliter plotted against PPO in years, including only amyloid‐positive subjects (*n* = 266). PPO was not a significant predictor of CSF p‐tau level in subjects evidenced to be on the Alzheimer's disease continuum by amyloid positivity, accounting for the effects of age, gender, and education level. Nor did APOE‐𝜀4 carriage significantly interact with PPO to predict CSF p‐tau level. Shaded areas indicate 95% confidence intervals for CSF marker levels.

Among amyloid‐positive subjects (*n* = 226), age (β = 0.61644; T = 6.945; *p* < 0.001) but not PPO (β = ‐0.055; T = ‐0.819; *p* = 0.414) significantly predicted CSF p‐tau level. PPO did not significantly interact with APOE‐𝜀4 status (Figure 4), nor age, gender, education, APOE‐𝜀2 status, family history load, risk inheritance, or CDR score to predict CSF p‐tau level (Table S3). The proportion of APOE‐𝜀4+/APOE‐𝜀2‐ subjects who were amyloid‐positive additionally meeting criteria for p‐tau positivity did not appear to rise substantially on approach of age of parental dementia onset (Figure 3). Post‐hoc analyses on a subsample whose parent had been diagnosed with dementia before age 85 (*n* = 195) did not show different results.

### 3.4 Cognitive performance as a function of PPO

Among amyloid‐positive subjects (*n* = 226), age was a significant predictor of 9 out of 13 ENE subscores. PPO was not a significant predictor of any of the subscores (Table S4). However, a trend toward significance was noted for the Favourites test subscore (*p* = 0.082) and education level interacted significantly with PPO to predict performance on this test (*p* = 0.030). Lower education level predicted a steeper decline in performance on approach of parental onset of dementia (Figure S2). PPO did not significantly interact with age, gender, APOE‐𝜀4 status, APOE‐𝜀2 status, family history load, risk inheritance, or CDR score to predict cognitive performance. It was noted that family history load interacted with PPO to predict RBANS Coding subscore with borderline significance (*p* = 0.058). Improved performance on approach of parental dementia onset observed in those with a dual parent history should be interpreted with caution due to the small size of this group (*n* = 26).

Post‐hoc analyses were again performed on a subsample whose parent had been diagnosed with dementia before age 85 (*n* = 195). In addition, analyses were conducted on the whole cohort to establish whether the results seen in amyloid positive subjects can be seen in those not evidenced to be on the AD continuum. Results are described in Supplementary Material (see Supplementary Results).

## 4 DISCUSSION

This study demonstrates that in APOE‐𝜀4 carriers PPO predicts accrual of amyloid pathology in mid‐to‐late life. Further, it indicates for the first time that the impact of APOE‐𝜀4 is dose‐dependent such that homozygosity predicts steeper progression of amyloid accrual over the same range of PPO values. The findings are consistent with a known impact of APOE‐𝜀4 on amyloid aggregation, deposition, and clearance from the brain^26^ and younger age of disease onset among APOE‐𝜀4 homozygotes.^24^ They also support genomic data suggesting that heritability of age of onset is largely driven by APOE.^10^, ^11^ It is of note that previous evidence of a stronger relationship between PPO and amyloid accrual in females^15^, ^16^ and during late middle life^15^ was not supported by the current study despite a much larger sample size.

Among those who are amyloid positive, PPO does not appear to predict further advancement along the disease continuum when indicated by CSF p‐tau. This study therefore substantiates the findings of Arenaza‐Urquijo and colleagues,^15^ additionally providing assurance that experimental noise created by individuals accruing tau in the absence of amyloid had not obscured an association relevant specifically to AD. Strikingly, it highlights the great potential for other factors to moderate disease trajectory even in APOE‐𝜀4 carriers.

PPO also did not convincingly predict cognitive decline among those who are amyloid positive. However, the trend‐level significance of PPO as a predictor of performance on a test of paired‐associate learning and the significant interaction between PPO and education level on this test were shown despite a small standard deviation from the mean score among the whole sample and are consistent with the known protective influence of education on cognition. Impaired paired‐associate learning has been previously associated with amyloid accrual,^27^, ^28^, ^29^ suggesting that subtle decline in episodic memory begins secondary to early molecular events rather than advancement of tauopathy.

It is important to note that the findings relate to time‐dependence of biomarker evidence, rather than overall risk. PPO is intended to reflect “when” events are likely to occur relative to a parent's age of onset, rather than “if” they will occur. Hence, results are not inconsistent with previous findings that amyloid positivity in APOE‐𝜀4 carriers is much more likely to progress to cognitive impairment than it is in non‐carriers.^30^ Instead, they suggest that in APOE‐𝜀4 carriers the timing of amyloidosis is more genetically preordained than the timing of disease advancement. It still follows that once the early “molecular driver”^31^ is present one is at risk of downstream pathology at a sooner time, perhaps explaining apparent heritability of age of symptomatic onset of LOAD.^9^, ^10^, ^11^ Presumably, a myriad of other genetic, epigenetic, and environmental protective and risk factors for AD modulate timing as well as likelihood of disease progression in a stochastic manner^31^ such that PPO cannot reliably predict tauopathy or early cognitive decline. Therein lies hope that, even when “Alzheimer's pathologic change”^23^ appears genetically predestined, one retains a degree of power to modify the course of LOAD.

Post‐hoc analyses suggesting that major modifiable risk factors for dementia do not significantly alter the relationship between PPO and amyloid accrual support that the foundation of the amyloid pathophysiological cascade is highly genetically driven. However, sizeable minorities of APOE‐𝜀4 carriers within 5 years of parental dementia onset and beyond parental dementia onset remained amyloid negative. This implies that other factors can still change early molecular events. Indeed, even in carriers of the same autosomal dominant mutation, there is some variability in disease course.^32^, ^33^

The results of the current study suggest that considering APOE‐𝜀4 carriers separately from non‐carriers in studies of preclinical LOAD could be more informative. It is possible that novel biological and cognitive changes previously correlated with a measure of temporal proximity to parental dementia onset^12^, ^13^, ^14^ have appeared weaker than they actually are or that relationships were obscured due to inclusion of both APOE‐𝜀4 carriers and non‐carriers, particularly in small samples. Corroborating this, Mak and colleagues^13^ evidenced early cerebral perfusion changes dependent on PPO only in APOE‐𝜀4 carriers. They also showed that PPO could predict white matter microstructural changes shown to precede grey matter atrophy^34^ but not grey matter volumes, supporting the idea that PPO is more sensitive to earlier pathological events.

Though larger samples will be required to clarify the impact of concomitant APOE‐𝜀2 carriage, it is noteworthy that, within the group of 25 APOE‐𝜀4+/ APOE‐𝜀2+ individuals included in this study, PPO was not a significant predictor of Aβ1‐42 level, suggesting that APOE‐𝜀2 provides a protective effect. Used exclusively in APOE‐𝜀4+/APOE‐𝜀2‐ individuals, PPO might be a powerful biomarker discovery tool by allowing elucidation of changes closely temporally related to amyloid accrual in LOAD without requiring amyloid‐specific blood, CSF, or positron emission tomography (PET) imaging tests.

Use of PPO as a risk stratification tool for amyloid positivity could have further diverse applications. In large prospective cohort studies and clinical trials requiring amyloid‐positive subjects, it could simplify recruitment, a simple history question helping to identify those at highest risk. In APOE‐𝜀4+ individuals, PPO also provides a method of identifying those who evade amyloid positivity despite numerically definable progression of risk, with opportunity to study characteristics that protect against early molecular events in the disease course. Further, following APOE‐𝜀4+/APOE‐𝜀2‐ individuals as they approach age of parental onset could explicate factors associated with swiftest or slowest disease progression after amyloid positivity. This could in turn help to answer the crucial question of which amyloid‐positive individuals are likely to progress to dementia within a given time frame, facilitating ethical testing of anti‐amyloid therapies in the early preclinical phase of AD. In the clinic, PPO could then inform the age at which APOE‐𝜀4+ individuals should receive amyloid screening. Finally, the relationships between PPO and AD markers in APOE‐𝜀4 carriers shown in the current study will facilitate genetic counseling of these individuals.

Limitations of this study include its cross‐sectional design, self‐reporting of parental dementia diagnosis, including date and unavailability of parental APOE status. It also used an indirect measure of cerebral amyloid, though an advantage of a CSF measure is that it may be more sensitive to preclinical AD.^35^, ^36^ While effort was made to isolate those on the AD spectrum for the second and third sets of analyses, this method may have excluded some subjects who were on the AD spectrum but did not yet meet the given threshold for amyloid positivity. Moreover, it treated amyloid accrual as a necessary criterion for AD, though this is controversial.^30^, ^37^ Finally, while the sample size used in this study conferred power to explore the impact of precise APOE‐𝜀 allele combination, it remained limited by the rarity of 2/4 and 2/2 combinations.

This study provides support for use of PPO as a method of stratifying risk of amyloid pathology in studies of LOAD. Its use as an empirical metric could help detect other incipient pathological changes paralleling or perhaps preceding amyloid accrual. The term *proximity to parental onset of dementia* (PPO) may be more appropriate than the previously utilized term *estimated years to onset of dementia* (EYO). Further, our results suggest that in studies of LOAD the metric is meaningfully applied only to APOE‐𝜀4 carriers and probably only to those not concomitantly carrying APOE‐𝜀2. As well as aiding biomarker discovery, the findings could be helpfully applied to trial recruitment, investigation of unknown risk and protective factors for disease progression, future amyloid screening protocols, and genetic counseling of APOE‐𝜀4 carriers. The study also highlights the amyloidagenic influence of APOE‐𝜀4, which could be exploited in drug discovery.

## CONFLICT OF INTEREST STATEMENT

Author Dag Aarsland has received research support and/or honoraria from Astra‐Zeneca, H. Lundbeck, Novartis Pharmaceuticals, Evonik, Roche Diagnostics, and GE Health, Sanofi, and served as a paid consultant for H. Lundbeck, Eisai, Heptares, Eli Lilly, Enterin, Acadia, EIP Pharma, Biogen, and Takeda. Author Craig Ritchie is the majority shareholder, founder and CEO of Scottish Brain Sciences. He has received consultancy fees from Biogen, Eisai, MSD, Actinogen, Roche, Virogenics, and Eli Lilly, as well as payment or honoraria from Roche and Eisai. Author John T. O'Brien has received support from Avid/Lilly, Merck, and Alliance Medical and has acted as consultant for TauRx, Novo Nordisk, Biogen, Roche, Lilly, and GE Healthcare. Authors Elijah Mak and Elina T. Ziukelis have no conflicts to disclose. Author disclosures are available in the Supporting Information.

## Supporting information



Supporting Information

Supporting Information
