# Supplementary material for: Accrual of Alzheimer's disease pathology as a function of proximity to parental dementia onset
Source: Alzheimers Dement (Amst). 2025 Feb 27;17(1):e70092. doi: 10.1002/dad2.70092 (PMC11865705; doi:10.1002/dad2.70092)
Supplement: Supplementary file 2 — Supporting Information [file DAD2-17-e70092-s002.docx]

**Supplementary Methods**

*Cerebrospinal Fluid Measures*

T-tau levels were not utilised in the current study because Ingala and colleagues [1] determined that p-tau levels are strongly linearly related to t-tau levels within the EPAD cohort. Therefore in this cohort t-tau levels may not be reflective of pathological transition to neurodegeneration according to the A/T/N framework.

*Cogntiive Testing*

The number of amyloid positive subjects for which the 13 included EPAD Neuropsychological Examination (ENE) subscores were available is as follows:

1. NIH Examiner Flanker test global score combining accuracy and reaction time (n=167)
2. RBANS Coding score (n=225)
3. RBANS List Learning score (n=225)
4. RBANS Story Memory score (n=225)
5. RBANS Figure Copy score (n=225)
6. RBANS Line Orientation score (n=225)
7. RBANS Picture Naming score (n=225)
8. RBANS Semantic Fluency score (n=225)
9. RBANS Digit Span score (n=225)
10. NIH Toolbox Dot Counting total number of correct responses across six trials (n=168)
11. University College London & Cambridge University Four Mountains Test total number of marks across six items (n=112)
12. NIH Toolbox Favourites test total number of correct responses summed across two learning trials and a delayed recall trial (n=160)
13. Cambridge University Virtual Reality Supermarket Trolley test total number of correct responses across fourteen trials (n=213).

**Supplementary Results**

*CSF Aβ1-42 as a function of Age*

Among the whole cohort (n=688), age was not a significant predictor of CSF Aβ1-42 level when PPO, gender and education were included as covariates (see Supplementary Table 1). Exploratory Spearman’s correlation analyses further suggest that PPO may become an increasingly better predictor of CSF Aβ1-42 level relative to age once individuals become amyloid positive (see Supplementary Figure 1).

*Cognitive performance as a function of PPO*

Post-hoc analyses were again performed on a subsample whose parent had been diagnosed with dementia before age 85 (n=195). In this group, age predicted only 7 ENE subscores. PPO was not a significant predictor of any of the subscores. Its trend-level significance as a predictor of Favourites performance disappeared (p=0.218). Significance of the interaction between PPO and education level in prediction of Favourites performance also disappeared (p=0.717) while the interaction between PPO and family history load in prediction of RBANS Coding performance became truly significant (p=0.010). No other interactions were identified.

Further post-hoc analyses were conducted in order to establish whether the results seen in amyloid positive subjects can be seen in those not evidenced to be on the AD continuum. In both the whole sample (n=688) and the amyloid negative group (n=462), each subscore was regressed on PPO including age as well as gender and education level as covariates. Interactions between PPO and education level and between PPO and family history load were also re-tested. Although when using the whole sample PPO became a truly significant predictor of Favourites performance (p=0.018), it was not a significant predictor among the amyloid negative group (p=0.495). PPO was not a significant predictor of any other test subscore in either the whole sample or the amyloid negative group. Nor did PPO interact with education level or family history load to predict any of the subscores in either group.

| **Independent Variable** | **β** | **T** | **p** |
| --- | --- | --- | --- |
| **Age** | **-2.911** | **-0.962** | **0.3364** |
| PPO | 9.357 | 4.161 | 3.58e-05 |
| Gender (Male) | -65.873 | -1.850 | 0.0648 |
| Education | -1.475 | -0.303 | 0.7620 |
| Adjusted R-squared: 0.04785 | | | |

**Supplementary Table 1: Age as a Predictor of Aβ1-42 among the whole cohort**

Table shows full results of the regression equation *CSF Aβ1-42 ~ Age + PPO + Gender + Education.*

| CSF marker | CSF Marker ~ Age^†^ | CSF Marker ~ PPO^‡^ | CSF Marker ~ PPO: Age^§^ | CSF Marker ~ PPO: Gender^§^ | CSF Marker ~ PPO: Education^§^ | CSF Marker ~ PPO: APOE-𝜀4 Status^§^ | CSF Marker ~ PPO: APOE-𝜀2 Status^§^ | CSF Marker ~ PPO: Family History Load^§^ | CSF Marker ~ PPO: Risk Inheritance^§^ | CSF Marker ~ PPO: CDR Total^§^ |
| --- | --- | --- | --- | --- | --- | --- | --- | --- | --- | --- |
| Aβ1-42 | **p<0.001***  ^(n=688)^ | **p<0.001***  ^(n=688)^ | p=0.779  ^(n=688)^ | p=0.949  ^(n=688)^ | p=0.883  ^(n=688)^ | E4+, **p=0.009***  E4++,  **p=0.021***  ^(n=654)^ | p=0.589  ^(n=654)^ | p=0.561  ^(n=688)^ | Pat,  p=0.774  Both,  p=0.609  ^(n=688)^ | p=0.183  ^(n=688)^ |
| **p<0.05*  *†Including gender and education level as covariates*  *‡Including age, gender and education level as covariates*  *§Including age, gender, education and interaction terms as covariates* | | | | | | | | | | |

**Supplementary Table 2: Prediction of Aβ1-42 among the whole cohort**

Table shows p values obtained during the first set of analyses. Where specified, *n* denotes the number of participants included in the regression model (limited in two cases by availability of APOE data).

| CSF marker | CSF Marker ~ Age^†^ | CSF Marker ~ PPO^‡^ | CSF Marker ~ PPO: Age^§^ | CSF Marker ~ PPO: Gender^§^ | CSF Marker ~ PPO: Education^§^ | CSF Marker ~ PPO: APOE-𝜀4 Status^§^ | CSF Marker ~ PPO: APOE-𝜀2 Status^§^ | CSF Marker ~ PPO: Family History Load^§^ | CSF Marker ~ PPO: Risk Inheritance^§^ | CSF Marker ~ PPO: CDR Total^§^ |
| --- | --- | --- | --- | --- | --- | --- | --- | --- | --- | --- |
| p-tau | **p<0.001***  ^(n=226)^ | p=0.414  ^(n=226)^ | p=0.579  ^(n=226)^ | p=0.923  ^(n=226)^ | p=0.862  ^(n=226)^ | E4+, p=0.791  E4++,  p= 0.751  ^(n=212)^ | p=0.614  ^(n=212)^ | p=0.976  ^(n=226)^ | Pat,  p=0.557  Both,  p=0.773  ^(n=226)^ | p= 0.519  ^(n=226)^ |
| **p<0.05*  *†Including gender and education level as covariates*  *‡Including age, gender and education level as covariates*  *§Including age, gender, education and interaction terms as covariates* | | | | | | | | | | |

**Supplementary Table 3: Prediction of CSF p-tau among amyloid positive subjects**

Table shows p values obtained during the second set of analyses. Where specified, *n* denotes the number of participants included in the regression model (limited in two cases by availability of APOE data).

| Cognitive Subtest | Score ~ Age^†^ | Score ~ PPO^‡^ | Score ~ PPO: Age^§^ | Score ~ PPO: Gender^§^ | Score ~ PPO: Education^§^ | Score ~ PPO: APOE-𝜀4 Status^§^ | Score ~ PPO: APOE-𝜀2 Status^§^ | Score ~ PPO: Family History Load^§^ | Score ~ PPO: Risk Inheritance^§^ | Score ~ PPO: CDR Total^§^ |
| --- | --- | --- | --- | --- | --- | --- | --- | --- | --- | --- |
| Flanker | **p<0.001***  ^(n=167)^ | p=0.961  ^(n=167)^ | p=0.989  ^(n=167)^ | p=0.943  ^(n=167)^ | p=0.249  ^(n=167)^ | E4+, p=0.868  E4++,  p=0.650  ^(n=163)^ | p=0.834  ^(n=163)^ | p=0.992  ^(n=167)^ | Pat,  p=0.970  Both,  p=0.959  ^(n=167)^ | p=0.857  ^(n=167)^ |
| Coding | **p<0.001***  ^(n=225)^ | p=0.790  ^(n=225)^ | p=0.989  ^(n=225)^ | p=0.943  ^(n=225)^ | p=0.249  ^(n=225)^ | E4+, p=0.868  E4++,  p=0.234  ^(n=211)^ | p=0.834  ^(n=211)^ | **p=0.058**  ^(n=225)^ | Pat,  p=0.970  Both,  p=0.124  ^(n=225)^ | p=0.857  ^(n=225)^ |
| List Learning | **p<0.001***  ^(n=225)^ | p=0.790  ^(n=225)^ | p=0.989  ^(n=225)^ | p=0.943  ^(n=225)^ | p=0.270  ^(n=225)^ | E4+, p=0.868  E4++,  p=0.234  ^(n=211)^ | p=0.721  ^(n=211)^ | p=0.759  ^(n=225)^ | Pat,  p=0.970  Both,  p=0.959  ^(n=225)^ | p=0.857  ^(n=225)^ |
| Story Memory | **p=0.010***  ^(n=225)^ | p=0.961  ^(n=225)^ | p=0.989  ^(n=225)^ | p=0.943  ^(n=225)^ | p=0.290  ^(n=225)^ | E4+, p=0.868  E4++,  p=0.234  ^(n=211)^ | p=0.721  ^(n=211)^ | p=0.759  ^(n=225)^ | Pat,  p=0.408  Both,  p=0.959  ^(n=225)^ | p=0.857  ^(n=225)^ |
| Figure Copy | p=0.575  ^(n=225)^ | p=0.961  ^(n=225)^ | p=0.989  ^(n=225)^ | p=0.943  ^(n=225)^ | p=0.917  ^(n=225)^ | E4+, p=0.868  E4++,  p=0.234  ^(n=211)^ | p=0.721  ^(n=211)^ | p=0.992  ^(n=225)^ | Pat,  p=0.408  Both,  p=0.959  ^(n=225)^ | p=0.857  ^(n=225)^ |
| Line Orientation | p=0.575  ^(n=225)^ | p=0.961  ^(n=225)^ | p=0.989  ^(n=225)^ | p=0.943  ^(n=225)^ | p=0.635  ^(n=225)^ | E4+, p=0.868  E4++,  p=0.375  ^(n=211)^ | p=0.633  ^(n=211)^ | p=0.992  ^(n=225)^ | Pat,  p=0.970  Both,  p=0.959  ^(n=226)^ | p=0.857  ^(n=225)^ |
| Picture Naming | p=0.842  ^(n=225)^ | p=0.774  ^(n=225)^ | p=0.989  ^(n=225)^ | p=0.964  ^(n=225)^ | p=0.781  ^(n=225)^ | E4+, p=0.868  E4++,  p=0.609  ^(n=211)^ | p=0.834  ^(n=211)^ | p=0.759  ^(n=225)^ | Pat,  p=0.970  Both,  p=0.959  ^(n=225)^ | p=0.857  ^(n=225)^ |
| Semantic Fluency | **p= 0.003***  ^(n=225)^ | p=0.538  ^(n=225)^ | p=0.989  ^(n=225)^ | p=0.964  ^(n=225)^ | p=0.781  ^(n=225)^ | E4+, p=0.868  E4++, p=0.234  ^(n=211)^ | p=0.834  ^(n=211)^ | p=0.759  ^(n=225)^ | Pat,  p=0.970  Both,  p=0.959  ^(n=225)^ | p=0.857  ^(n=225)^ |
| Digit Span | p=0.487  ^(n=225)^ | p=0.961  ^(n=225)^ | p=0.920  ^(n=225)^ | p=0.964  ^(n=225)^ | p=0.635  ^(n=225)^ | E4+, p=0.868  E4++, p=0.234  ^(n=211)^ | p=0.940  ^(n=211)^ | p=0.992  ^(n=225)^ | Pat,  p=0.970  Both,  p=0.959  ^(n=225)^ | p=0.857  ^(n=225)^ |
| Dot Counting | **p=0.004***  ^(n=168)^ | p=0.961  ^(n=168)^ | p=0.989  ^(n=168)^ | p=0.964  ^(n=168)^ | p=0.781  ^(n=168)^ | E4+, p=0.868  E4++, p=0.459  ^(n=164)^ | p=0.834  ^(n=164)^ | p=0.992  ^(n=168)^ | Pat,  p=0.970  Both,  p=0.959  ^(n=168)^ | p=0.857  ^(n=168)^ |
| Four Mountains Test | **p<0.001***  ^(n=112)^ | p=0.961  ^(n=112)^ | p=0.920  ^(n=112)^ | p=0.964  ^(n=112)^ | p=0.781  ^(n=112^ | E4+, p=0.868  E4++, p=0.717  ^(n=106)^ | p=0.721  ^(n=106)^ | p=0.992  ^(n=112)^ | Pat,  p=0.970  Both,  p=0.962  ^(n=112)^ | p=0.857  ^(n=112)^ |
| Favourites | **p=0.002***  ^(n=160)^ | **p=0.082**  ^(n=160)^ | p=0.989  ^(n=160)^ | p=0.943  ^(n=160)^ | **p=0.030***  ^(n=160)^ | E4+, p=0.868  E4++, p=0.234  ^(n=156)^ | p=0.721  ^(n=156)^ | p=0.992  ^(n=160)^ | Pat,  p=0.970  Both,  p=0.959  ^(n=160)^ | p=0.857  ^(n=160)^ |
| Virtual Reality Supermarket Trolley | **p<0.001***  ^(n=213)^ | p=0.538  ^(n=213)^ | p=0.920  ^(n=213)^ | p=0.964  ^(n=213)^ | p=0.957  ^(n=213)^ | E4+, p=0.868  E4++, p=0.459  ^(n=199)^ | p=0.834  ^(n=199)^ | p=0.992  ^(n=213)^ | Pat,  p=0.970  Both,  p=0.959  ^(n=213)^ | p=0.857  ^(n=213)^ |
| **p<0.05*  *†Including gender and education level as covariates*  *‡Including age, gender and education level as covariates*  *§Including age, gender, education and interaction terms as covariates* | | | | | | | | | | |

**Supplementary Table 4: Prediction of EPAD Neuropsychological Examination (ENE) subscores among amyloid positive subjects**

Table shows p values obtained during the third set of analyses after correction for multiple comparisons. Where specified, *n* denotes the number of participants included in the regression model (limited in some cases by availability of APOE data and specific cognitive test scores). Age predicted performance on a majority of the included cognitive test subscores and across all domains except for visuospatial analysis. Proximity to parental dementia onset (PPO) did not predict performance on any of the subscores, although the p value for the ‘Favourites’ subscore was of trend level significance and education level interacted significantly with PPO to predict it.


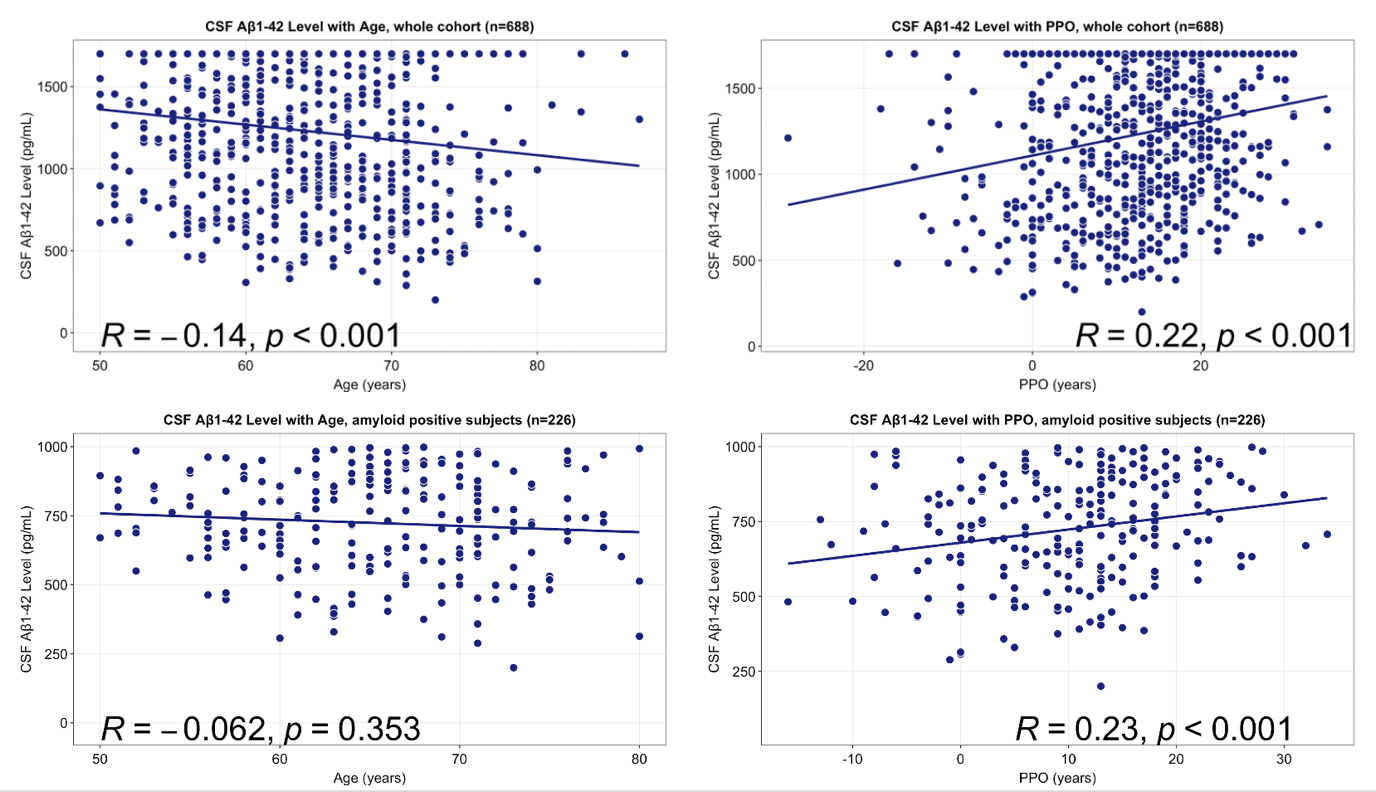


**Supplementary Figure 1: Associations between CSF Aβ1-42 level, age and PPO among the whole cohort and among amyloid positive subjects**

Shows results of Spearmans Rank Correlation analyses. Among the whole cohort (n=688), both age (top left) and PPO (top right) were significantly associated with CSF Aβ1-42 level. However, among amyloid positive subjects (n=226), PPO (bottom right) but not age (bottom left) was significantly associated with CSF Aβ1-42 level.

**Supplementary Figure 2: Paired-associate learning performance as a function of PPO in amyloid positive subjects**

Total number of correct responses summed across two learning trials and a delayed recall trial during the ‘Favourites’ paired-associate learning task plotted against proximity to parental onset of dementia (PPO). Although PPO predicted performance with only trend-level significance (p=0.082), education level interacted significantly with PPO to predict performance (p=0.030), a lower level of education predicting steeper decline in performance. Among the sample for which scores were available (n=160), mean performance was 6.875±1.491 (range 1-8). Shaded areas indicate 95% confidence intervals for test subscores.

**References**

[1] Ingala S, De Boer C, Masselink LA, Vergari I, Lorenzini L, Blennow K, Chételat G, Di Perri C, Ewers M, van der Flier WM, Fox NC, Gispert JD, Haller S, Molinuevo JL, Muniz-Terrera G, Mutsaerts HJ, Ritchie CW, Ritchie K, Schmidt M, Schwarz AJ, Vermunt L, Waldman AD, Wardlaw J, Wink AM, Wolz R, Wottschel V, Scheltens P, Visser PJ, Barkhof F; EPAD consortium. Application of the ATN classification scheme in a population without dementia: Findings from the EPAD cohort. Alzheimers Dement. 2021 Jul;17(7):1189-1204.
